# Knowledge, Attitudes and Practices of Japanese Cardiovascular Surgeons on the Management of Surgical Site Complications and Prophylactic Single-Use Negative Pressure Wound Therapy After Coronary Artery Bypass Surgery: A Quantitative Survey

**DOI:** 10.7759/cureus.87497

**Published:** 2025-07-08

**Authors:** Kazuyoshi Takagi, Yan Ran Wee, Natsumi Fujita, Alvin Ng, Seth Francis-Graham, Akie Seno

**Affiliations:** 1 Division of Cardiovascular Surgery, Department of Surgery, Kurume University, Kurume, JPN; 2 Evidence Development, Costello Medical, Singapore, SGP; 3 Health Policy, Costello Medical, London, GBR; 4 Market Access Asia Cluster, Smith and Nephew, Singapore, SGP

**Keywords:** coronary artery bypass grafting, negative pressure wound therapy, reimbursement, surgical site complication, surgical site infection

## Abstract

Objective

This study assessed the knowledge, attitudes and practices of cardiovascular surgeons in Japan on prophylactic single-use negative pressure wound therapy (sNPWT) after coronary artery bypass grafting (CABG).

Methods

A survey was distributed online to an anonymous Japanese market research panel in April 2024 (target: 50 responses). Eligible respondents were cardiovascular surgeons practising in Japan, with five or more years of experience and having performed 20 or more CABG surgeries in the past year. Responses were analysed descriptively.

Results

Among the 50 respondents (38% general, 30% national/public, 24% university hospitals), most agreed that the consequences of surgical site complications (SSCs) following cardiovascular surgery were more severe than other surgeries (78%), that SSC prevention is a priority after CABG (80%), and that postoperative procedures are important for managing SSC risk (84%). However, sNPWT was rarely used routinely (2%). Only 24% reported prophylactic sNPWT use; 44% used sNPWT only upon signs of SSCs. Cost-related factors were important to 60‒70% of respondents in determining SSC prevention. There was low knowledge of approved indications and reimbursement criteria for sNPWT. While 52% would recommend prophylactic sNPWT, limited reimbursement (80%) and insufficient familiarity with safety (52%) and effectiveness (50%) data were reasons for non-use. Sixty percent of respondents agreed that Japan-specific sNPWT guidelines are needed.

Conclusions

Barriers to using prophylactic sNPWT in Japan include complex and limited reimbursement criteria, cost concerns, insufficient local evidence and low awareness of existing evidence. Addressing these challenges may encourage prophylactic sNPWT use, leading to reduced postoperative complications, lower healthcare costs, and minimised disparities in care.

## Introduction

Surgical site complications (SSCs), including wound dehiscence, seroma and surgical site infections (SSIs), can occur on closed surgical incisions. Prevention of SSCs during post-operative management helps to avoid undesirable outcomes, such as delayed wound healing and abnormal scarring of the incision wound [1]. This is particularly important for some cardiovascular (CV) surgeries, including coronary artery bypass grafting (CABG), left ventricular assist device surgeries and transplant surgeries, where SSCs and their consequences can be particularly severe [1].

CABG is recognised as a particularly high-risk procedure, due to the potential for severe SSCs like sternal wound infections (SWIs), which are associated with an increased risk of mortality, prolonged hospital stays and are a significant treatment resource burden [2,3]. Data from the Japan Adult Cardiovascular Surgery Database demonstrated that among 46,268 patients who received CABG during the study period (2008-2011), 1.9% (n=859) were diagnosed with deep SWIs, with substantial variation across hospitals in Japan [4].

The risk of SSCs after CABG is also dependent on the surgical approach taken; conventional CABG performed through a midline sternotomy is associated with higher complication rates compared to minimally invasive CABG [5]. Global data has also shown that SSIs at secondary incision sites such as the saphenous vein harvest site can be a substantial source of morbidity, particularly with the use of conventional vein harvesting techniques, compared with newer minimally invasive approaches which remain less common [6,7]. In Japan, the vast majority (94.2%) of CABG procedures in 2019 used saphenous vein grafts (SVGs) or internal thoracic artery grafts (ITAGs) [8]. As previous research has suggested that perioperative treatment procedures, including surgical wound management, may be a primary factor affecting the risk of hospital-acquired infections, such as SSIs after CV surgery [4,9], incorporating prophylactic measures following high-risk surgeries like CABG could help minimise post-operative complications [10].

Negative pressure wound therapy (NPWT) systems, including single-use NPWT (sNPWT), can promote healing on closed surgical incisions by reducing lateral tension and improving lymphatic clearance (reducing the formation of oedema, seroma and haematomas) [11]. When used prophylactically on closed surgical incisions, meta-analyses have shown that sNPWT can reduce the incidence of SSCs compared to standard of care (SoC) [12-14]. In addition to clinical benefits, use of NPWT on closed surgical incisions can alleviate the economic burden on healthcare systems by reducing the length of hospital stays and re-operation rates [12,13].

International guidelines have been published on the use of NPWT and its role in reducing the occurrence of SSCs, particularly in high-risk patients. The World Health Organization’s 2018 Global Guidelines for the Prevention of Surgical Site Infection suggest that NPWT should be used prophylactically, primarily on closed surgical incisions in high-risk wounds, for the purpose of SSI prevention [15]. The World Union of Wound Healing Society (WUWHS) also published a 2016 consensus article on the role of NPWT in closed surgical incision management, highlighting that the assessment of SSC risk should take into account both the incidence and the severity of SSCs associated with each surgical procedure [1]. The article proposed that NPWT should be used prophylactically on closed surgical incisions for patients who have risk factors for SSCs, as well as those who are undergoing surgical procedures like CABG, which are associated with higher incidence and/or higher consequences of SSCs [1].

In Japan, sNPWT was approved in 2014 by the Ministry of Health, Labour and Welfare (MHLW) for use in refractory open wounds [16]. In 2019, the approved indication was expanded to include use on closed surgical wounds in patients at high SSI risk [17]. However, sNPWT is currently reimbursed under the National Health Insurance (NHI) system only in much narrower indications, where patients must fulfil two sets of criteria: (1) care setting: patients must be treated in the intensive care unit (ICU), stroke care unit, paediatric ICU, neonatal ICU or perinatal ICU; (2) high-risk definition: patients much have at least one of the NHI definitions of high risk (obesity with a body mass index of ≥30, diabetes with a glycated haemoglobin count of ≥6.6% or ≥7.0%, receiving steroid-based treatments, receiving chronic dialysis treatments, immunocompromised, malnourished, skin or cutaneous blood flow diseases or disorders that delay wound healing, or undergoing re-surgery at the same site as a prior surgery).

In the context of this complex reimbursement criteria and the lack of Japan-specific consensus articles or guidelines on the prophylactic use of NPWT following CV surgery, the current use of NPWT after CV surgery in clinical practice in Japan remains unclear. Therefore, this study conducted a survey to assess the knowledge, attitudes and practices of experienced CV surgeons in Japan. Key objectives were to understand current awareness and perceptions of SSC risk, as well as gain insights on CV surgeons’ knowledge of and attitudes on SSC preventative measures and management strategies, including sNPWT, after CABG. We then also sought to investigate potential barriers to sNPWT use. Some results from this study were previously presented as an oral presentation at the 55th Annual Meeting of the Japanese Society for Cardiovascular Surgery on February 20, 2025.

## Materials and methods

Targeted searches

Targeted Google searches were performed to identify relevant clinical studies, economic studies, guidelines and consensus papers published up to August 2023. Google was used as guidelines published in grey literature (e.g., medical society websites) may not be indexed in medical literature databases such as MEDLINE. Identified guidelines, consensus papers and clinical or economic studies were assessed for relevance based on the intervention of interest being sNPWT. The findings from the search were used to draft survey questions, which were then finalised based on feedback from a Japanese clinical expert.

Study design

A quantitative cross-sectional market research survey was conducted in April 2024. It was distributed via an online survey platform to a proprietary panel comprising users from m3.com, a website with over 900,000 users across medical specialties and membership by 90% of doctors in Japan. Screening questions were used to identify eligible participants who: (1) were CV surgeons, licenced and actively practicing in Japan; (2) had over five years of experience; (3) had performed at least 20 CABG surgeries using SVGs or ITAGs in the past year; (4) provided written informed consent to participate in the survey. The target number of respondents was 50, and recruitment stopped once the target was reached via convenience sampling.

The survey contained 35 multiple-choice questions in Japanese and consisted of the following sections (the full English-language questionnaire, prior to translation, can be found in Appendices): (1) screening and consent; (2) SSCs and incision management: thoughts on, and personal experiences with, SSCs, and current approach to prevention and management of SSCs; (3) current practices and perceptions of sNPWT: knowledge, perceptions and current usage patterns of sNPWT; (4) evidence base and current consensus on sNPWT: understanding of the evidence available on sNPWT and the current clinical guidelines or expert consensus on NPWT, and the extent to which these may affect clinical decision-making; (5) demographics. The content of the questionnaire was developed in collaboration with a cardiovascular surgeon to ensure appropriateness and relevance.

All responses were anonymised by the market research organisation. The investigators and sponsors of the study were blinded to the respondents’ identities. This market research study was sponsored by Smith & Nephew. Respondents were not informed of the identity of the study sponsor until after the completion of the survey.

The questionnaire was pre-tested in a pilot survey of 10% of the target sample (n=5), where respondents were invited to complete the survey and provide open-ended feedback on the structure, question wording, ease of use and time required for completion. Based on the responses received, the survey questions and response options were refined before the full launch. Responses from the pilot survey were not included in the final analysis, due to differences in the questions and answer options available.

Analysis

Results were summarised descriptively by number and percentage, as well as mean, median, standard deviation (SD) and interquartile range for continuous variables.

For questions designed to assess respondents’ knowledge of a topic, “knowledge scores” were calculated, with points awarded for either selecting all the correct (and no wrong) options, or selecting more right than wrong options. Aggregate scores were calculated across knowledge questions to assess the respondents’ overall knowledge, where a higher score indicated greater knowledge (minimum‒maximum score: 0.00‒1.00). For questions designed to assess respondents’ agreement with a position or statement, “strength of agreement scores” were calculated for each relevant Likert scale question (strongly disagree = 0; strongly agree = 100). Scores were aggregated by topic, where a higher score indicated greater agreement with a position or statement. Chronbach’s alpha was used to measure the internal consistency and reliability of Likert scale questions within the questionnaire, where higher scores indicated greater consistency and reliability.

Subgroup analyses were conducted, stratifying respondents by their sNPWT usage patterns: (1) routine prophylactic sNPWT use; (2) sNPWT use only upon signs of an SSC; (3) sNPWT use in the past but not currently; (4) never used sNPWT.

## Results

Invitations to participate in the survey were sent to over 40,000 cardiovascular, ICU and general surgeons, and 84% of respondents to the invitation were screened out due to not meeting the eligibility criteria. The survey was closed upon reaching the target enrolment of 50 responses. However, during data quality checks, six responses were removed due to quality concerns (e.g. completion time of less than five minutes or repeatedly selecting matrix choice questions in a single straight line). Survey recruitment was reopened briefly to replace these six respondents and acquire the final analysis dataset of 50 surgeons.

Of the 50 respondents in the final analysis dataset, 98% identified as male (n=49) (Table 1). The most common age group was 40-49 (42%, n=21) years, followed by 50-59 years (38%, n=19). Respondents from different types of hospitals were included, with 38% (n=19) from general hospitals, 30% (n=15) from national/public hospitals, and 24% (n=12) from university hospitals. All respondents were highly-experienced CV surgeons: 78% had over 20 years of experience (n=39), 40% were Chairs of their department (n=20), and half had performed more than 30 CABG surgeries in the past year (50%, n=25).

**Table 1:** Demographic characteristics of survey respondents Abbreviations: CABG: coronary artery bypass grafting; sNPWT: single-use negative pressure wound therapy; SSC: surgical site complication.

	All respondents (N=50)	Routine Prophylactic sNPWT users (N=12)	sNPWT users only upon signs of SSCs (N=22)	Past sNPWT users (N=6)	sNPWT never users (N=10)
	n (%)	n (%)	n (%)	n (%)	n (%)
Gender					
Male	49 (98%)	11 (92%)	22 (100%)	6 (100%)	10 (100%)
Female	0 (0%)	0 (0%)	0 (0%)	0 (0%)	0 (0%)
Prefer not to say	1 (2%)	1 (8%)	0 (0%)	0 (0%)	0 (0%)
Age (years)					
30–39	4 (8%)	1 (8%)	1 (5%)	1 (17%)	1 (10%)
40–49	21 (42%)	5 (42%)	7 (32%)	4 (67%)	5 (50%)
50–59	19 (38%)	4 (33%)	10 (45%)	1 (17%)	4 (40%)
≥60	6 (12%)	2 (17%)	4 (18%)	0 (0%)	0 (0%)
Years in practice					
5–9	1 (2%)	0 (0%)	0 (0%)	1 (17%)	0 (0%)
10–15	10 (20%)	4 (33%)	4 (18%)	1 (17%)	1 (10%)
≥20	39 (78%)	8 (67%)	18 (82%)	4 (67%)	9 (90%)
Number of CABG surgeries in past year					
20–29	25 (50%)	7 (58%)	11 (50%)	3 (50%)	4 (40%)
≥30	25 (50%)	5 (42%)	11 (50%)	3 (50%)	6 (60%)
Type of hospital					
University	12 (24%)	7 (58%)	2 (9%)	2 (33%)	1 (10%)
National/public	15 (30%)	3 (25%)	7 (32%)	2 (33%)	3 (30%)
Other public	4 (8%)	0 (0%)	4 (18%)	0 (0%)	0 (0%)
General	19 (38%)	2 (17%)	9 (41%)	2 (33%)	6 (60%)
Professional role					
Chair of department	20 (40%)	2 (17%)	11 (50%)	4 (67%)	3 (30%)
Director of department	7 (14%)	0 (0%)	3 (14%)	0 (0%)	4 (40%)
Chief physician	12 (24%)	4 (33%)	5 (23%)	1 (17%)	2 (20%)
Senior physician	5 (10%)	2 (17%)	1 (5%)	1 (17%)	1 (10%)
Senior consultant	5 (10%)	3 (25%)	2 (9%)	0 (0%)	0 (0%)
Other	1 (2%)	1 (8%)	0 (0%)	0 (0%)	0 (0%)

Perceptions of SSCs in CV surgery

**Figure 1:**
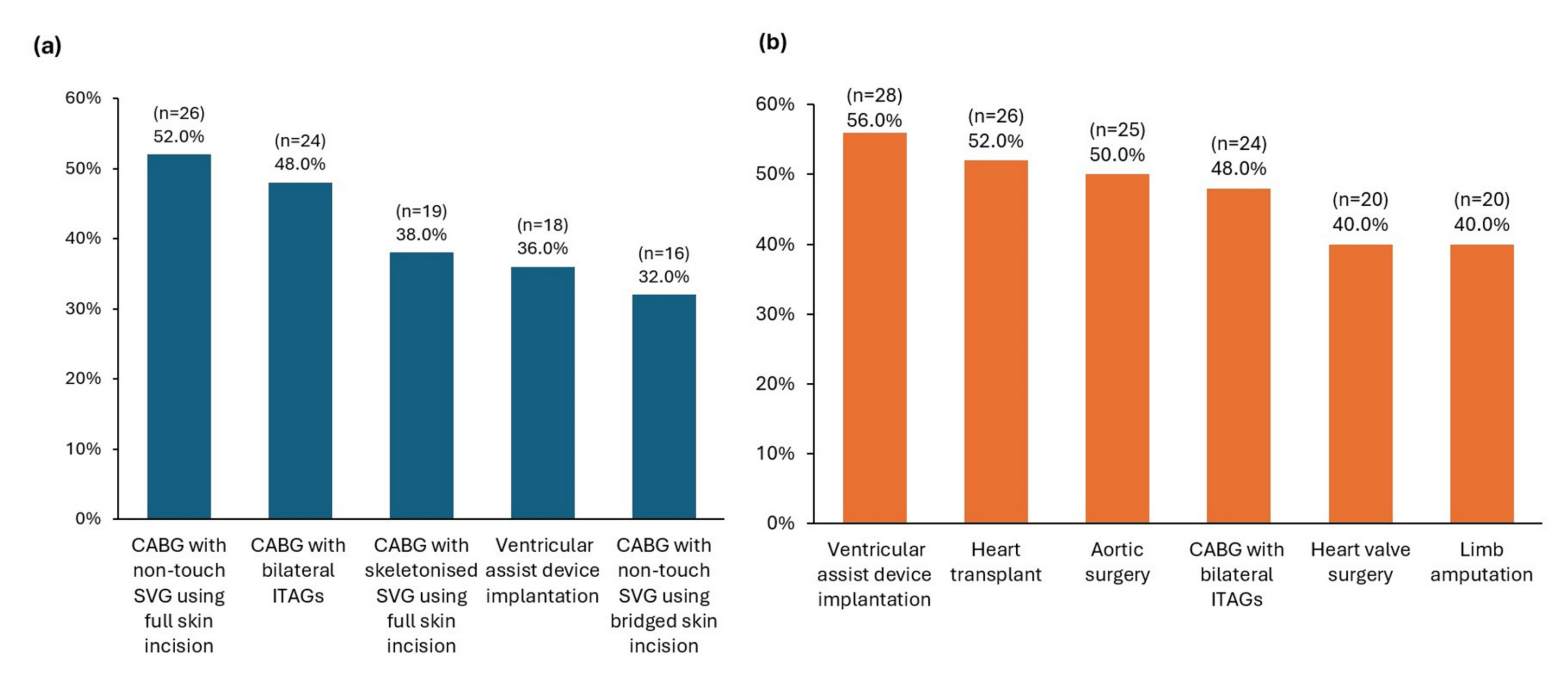
Perceptions of (a) SSC risk and (b) severity of consequences by CV surgeons, for different types of CV surgery among survey respondents (N=50) Footnotes: Respondents were asked: “For each of the following cardiovascular surgery types, please indicate if they may be associated with higher likelihood of SSCs or more severe consequences of SSCs, compared with other types of surgery.” Abbreviations: CABG: coronary artery bypass grafting; CV: cardiovascular; ITAG: internal thoracic artery graft; SSC: surgical site complication; SVG: saphenous vein graft.

Responses on the likelihood of an SSC following CV surgery, compared with other surgery types, were mixed. While 44% (n=22) of respondents reported a lower likelihood of SSCs following CV surgery, 24% (n=12) thought the likelihood of SSCs was higher and 32% (n=16) reported an average likelihood of SSCs. Among CV surgery types, CABG with non-touch SVG using full skin incision (52%, n=26), CABG with bilateral ITAGs (48%, n=24) and CABG with skeletonised SVG using full skin incision (38%, n=19) were most commonly indicated to have a higher likelihood of SSCs (Figure 1A).

However, most respondents (78%, n=39) agreed that the consequences of SSCs following CV surgeries are more severe than in other surgery types (consequences are the same: 18% [n=9]; less severe: 4% [n=2]). The types of CV surgery most commonly perceived to have more severe consequences were: ventricular assist device implantation (56%, n=28), heart transplants (52%, n=26) and aortic surgery (50%, n=25) (Figure 1B).

Management of SSCs after CABG

SSCs were reportedly uncommon, with 22% (n=11) of respondents having encountered no patients with SSCs in the past five years. About half of respondents had managed one to five patients with SSCs in the median sternotomy (50%, n=25) and lower extremity (54%, n=27) in the past five years. The majority (80%, n=40) of respondents agreed or strongly agreed that the prevention of SSCs (including SSIs) is a priority after CABG (16% neutral; 4% disagreed); the mean±SD strength of agreement score was 79.60±14.84 (maximum: 100).

**Figure 2:**
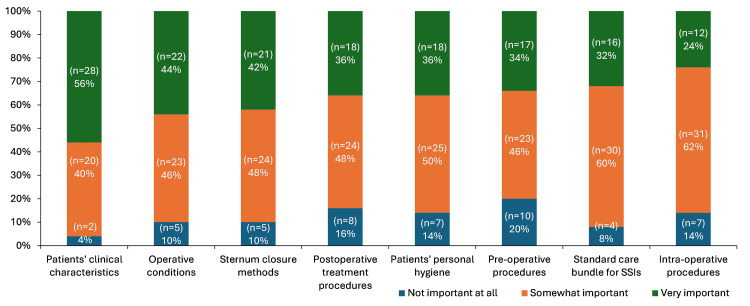
Perceptions of the importance of factors that contribute to SSC risk after CABG among survey respondents (N=50) Footnotes: Respondents were asked: “Please rate the importance of the following factors in contributing to the risk of a patient experiencing SSCs after CABG surgery.” Abbreviations: CABG: coronary artery bypass grafting; SSC: surgical site complication; SSI: surgical site infection.

While patients’ clinical characteristics were rated as the most important factor in contributing to SSC risk post-CABG (96% [n=48] somewhat or very important), 84% (n=42) also believed that postoperative procedures were somewhat or very important in managing SSC risk (Figure 2).

**Figure 3:**
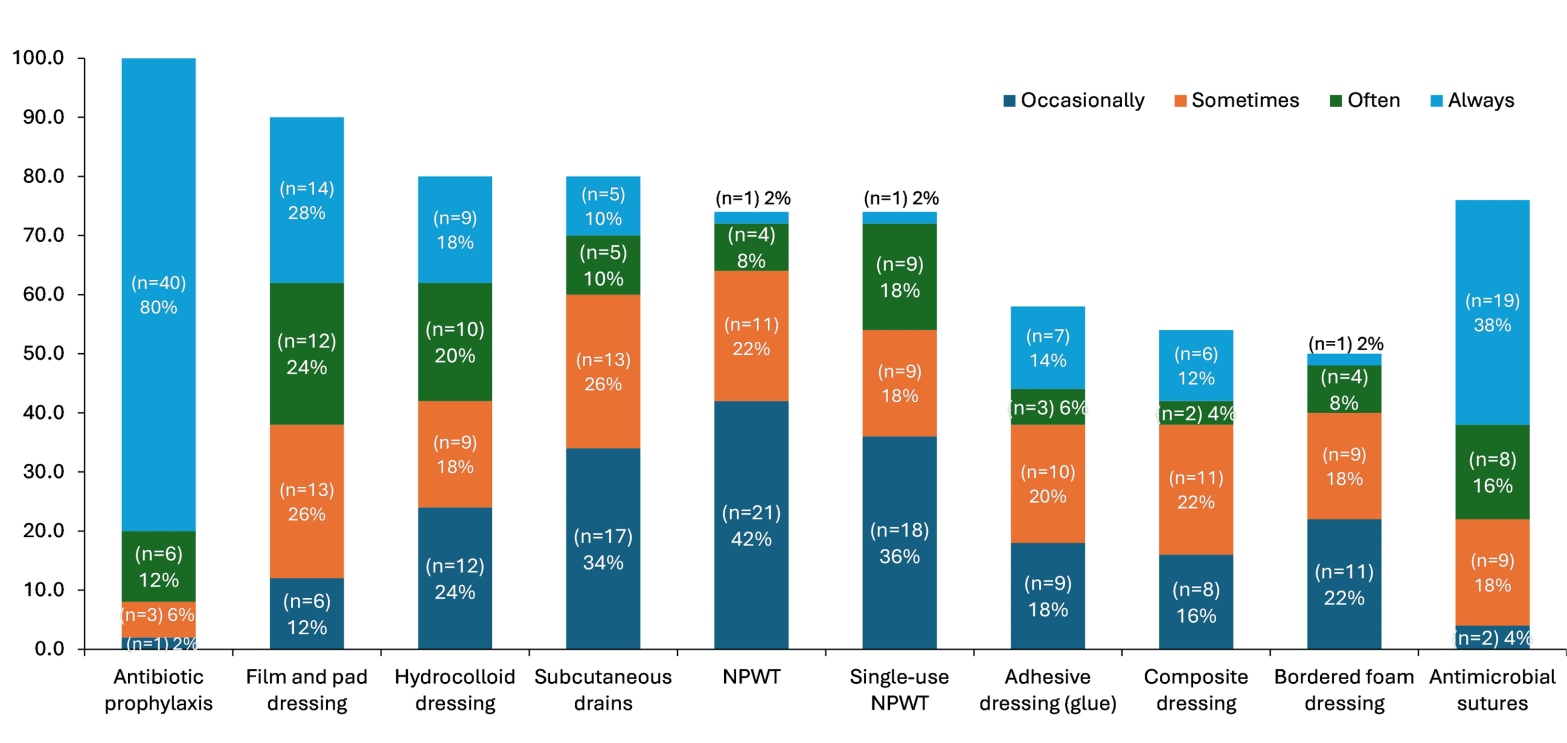
Current practices for SSC prophylaxis after CABG among survey respondents (N=50) Footnotes: Respondents were asked: “Which of the following strategies do you currently use to prevent the occurrence of SSCs, including SSIs in your patients, after CABG surgery?” Abbreviations: CABG: coronary artery bypass grafting; NPWT: negative pressure wound therapy; SSC: surgical site complication.

Antibiotics were the most common SSC prevention strategy, with 80% (n=40) of surgeons “always” prescribing them to patients after CABG surgery (Figure 3). Film and pad dressings were “always” or “often” used by 52% (n=26) of surgeons, whereas NPWT or sNPWT were “always” used by 2% (n=1) of surgeons.

Current practices and perceptions of sNPWT

Only 30% (n=15) of surgeons reported that they were “very familiar” with sNPWT and 62% (n=31) responded that they were “somewhat familiar”. All respondents who reported being “unsure” (4%, n=2) or “not at all familiar” (4%, n=2) with sNPWT had never used sNPWT.

**Figure 4:**
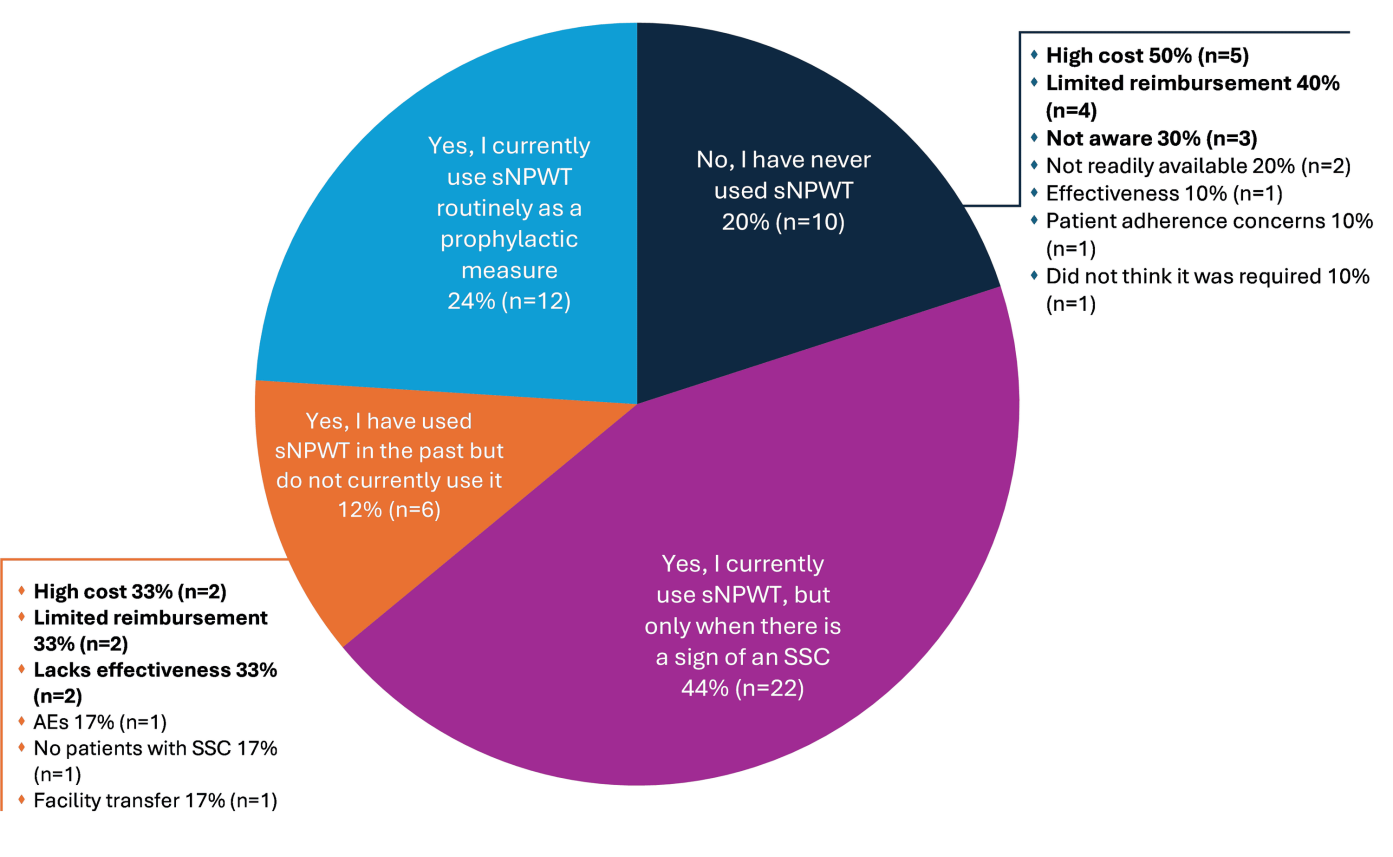
Current use of prophylactic sNPWT after CABG and reasons for non-use among survey respondents (N=50) Footnotes: Respondents were asked: “Have you ever used sNPWT on closed surgical wounds after conventional CABG with open SVG/ITAG harvesting?” Abbreviations: AE: adverse event; CABG: coronary artery bypass grafting; sNPWT: single-use negative pressure wound therapy; SSC: surgical site complication.

Only 24% (n=12) of surgeons used sNPWT routinely as a prophylactic measure, with nearly half the surgeons only using it when there was a sign of an SSC (44%, n=22) (Figure 4). Ten respondents had used sNPWT in other surgery types (emergency open chest surgery, mediastinitis surgery, CABG follow-up surgery, bypass of lower limbs, vacuum-assisted closure, ventricular assist device attachment, heart surgery, great vessels, open heart surgery with long-term cardiopulmonary ventilation). Reasons for not using sNPWT among never users and past users included high cost (44%; n=7/16) and limited reimbursement (38%; n=6/16).

There was low knowledge of the approved indications for sNPWT in Japan; the mean±SD knowledge score was 0.12±0.33 out of 1.00. Just 8% (n=4) of respondents selected all the correct approved indications. There were two respondents (4%) who selected “none of the above”, and the most commonly omitted approved indication was traumatic wounds (missed by 80%, n=40) (Table 2).

**Table 2:** Approval indications for sNPWT in Japan, as selected by survey respondents (N=50) Footnotes: ^*^Correct answers for knowledge questions regarding approval by the MHLW. Respondents were asked “Based on your understanding, please select all indications for which sNPWT is approved for use in Japan.” Abbreviations: MHLW: Ministry of Health, Labour and Welfare; SSC: surgical site complication; SSI: surgical site infection; sNPWT: single-use negative pressure wound therapy.

Approved indications	All respondents, n (%)		
Closed surgical wounds in patients at high SSI risk for prophylactic purpose^*^	36 (72%)		
Hard-to-heal open wounds^*^	35 (70%)		
Closed surgical wounds to treat SSCs^*^	31 (62%)		
Traumatic wounds^*^	10 (20%)		
None of the above	2 (4%)		

Respondents also had low knowledge of the care settings reimbursed under the NHI for prophylactic use of sNPWT in Japan. No respondent selected all the correct care settings for reimbursement in Japan (mean±SD knowledge score: 0.00±0.00) (Table 3). “ICU” was correctly selected by 94% (n=47) of respondents. “General ward” and “emergency treatment” were incorrectly selected by 52% (n=26) and 46% (n=23) of respondents, respectively.

**Table 3:** Reimbursed care settings for sNPWT in Japan, as selected by survey respondents (N=50) Footnotes: ^*^Correct answers for knowledge questions regarding reimbursement by the NHI. Respondents were asked “sNPWT was approved in Japan for prophylactic use in closed surgical wounds in patients at high SSI risk in 2019. However, Japan reimbursement criteria specify that sNPWT will be reimbursed for closed surgical wounds under the NHI system only in a subset of ‘high-risk’ patients, and only in specific care settings. Based on your understanding, please select the settings in which sNPWT may be reimbursed.” Abbreviations: ICU: intensive care unit; NHI: National Health Insurance; SSI: surgical site infection; sNPWT: single-use negative pressure wound therapy.

Reimbursed care settings	All respondents, n (%)		
ICU^*^	47 (94%)		
High care unit^*^	24 (48%)		
Neonatal ICU^*^	5 (10%)		
Paediatric ICU^*^	5 (10%)		
Stroke care unit^*^	4 (8%)		
Perinatal ICU^*^	4 (8%)		
General ward	26 (52%)		
Emergency treatment	23 (46%)		

However, the knowledge of the indications reimbursed under the NHI was higher (mean±SD knowledge score: 0.92±0.27). Among the 14 incorrect answers, 48% (n=24) of respondents selected “patients with diabetes using insulin” rather than the correct indication (“patients with HbA1C count ≥6.6%”), which was only selected by 38% (n=19) of the respondents (Table 4). Among the eight correct answers, the least known indications were “patients undergoing re-surgery at the same site as a prior surgery”, selected by 26% (n=13) of respondents, and “patients who are undernourished”, selected by 30% (n=15) of respondents.

**Table 4:** Reimbursed indications for sNPWT in Japan, as selected by survey respondents (N=50) Footnotes: ^*^Correct answers for knowledge questions regarding reimbursement by the NHI. Respondents were asked “From the following indications, please select all that apply for prophylactic use of sNPWT (1) indication for sNPWT covered by NHI reimbursement; (2) indication for sNPWT at my own hospital or institution; (3) indication that I hope to see sNPWT expanded to in the future; (4) not a suitable indication for sNPWT.” Abbreviations: BMI: body mass index; COPD: chronic obstructive pulmonary disease; ITAG: internal thoracic artery graft; NHI: National Health Insurance; SSC: surgical site complication; sNPWT: single-use negative pressure wound therapy.

Reimbursed indications	All respondents, n (%)		
	Indication for sNPWT covered by NHI	Indication for sNPWT at my own hospital or institution	Indication I hope to see sNPWT expanded to in the future
Patients with obesity (BMI ≥30)^*^	24 (48%)	16 (32%)	9 (18%)
Patients who are undernourished^*^	15 (30%)	14 (28%)	15 (30%)
Patients with diabetes (glycated haemoglobin count ≥6.6%)^*^	19 (38%)	16 (32%)	14 (28%)
Patients on dialysis^*^	23 (46%)	16 (32%)	11 (22%)
Patients on steroid based treatments^*^	26 (52%)	15 (30%)	12 (24%)
Patients who are immunocompromised^*^	24 (48%)	16 (32%)	14 (28%)
Patients with skin or cutaneous blood flow diseases and disorders that delay wound healing^*^	16 (32%)	18 (36%)	11 (22%)
Patients undergoing re-surgery at the same site as a prior surgery^*^	13 (26%)	10 (20%)	11 (22%)
Patients above the age of 65	9 (18%)	8 (16%)	12 (24%)
Patients above the age of 80	15 (30%)	13 (26%)	11 (22%)
Patients who are underweight (BMI <18)	9 (18%)	7 (14%)	11 (22%)
Patients with diabetes using insulin	24 (48%)	18 (36%)	13 (26%)
Patients with chronic kidney disease	13 (26%)	12 (24%)	14 (28%)
Patients who are current smokers	5 (10%)	10 (20%)	14 (28%)
Patients with COPD	11 (22%)	13 (26%)	15 (30%)
Patients with dementia	4 (8%)	3 (6%)	9 (18%)
Patients on blood-thinning medications	4 (8%)	3 (6%)	13 (26%)
Patients with a history of SSCs	12 (24%)	13 (26%)	13 (26%)
Patients undergoing emergency surgery	11 (22%)	8 (16%)	16 (32%)
Patients with bilateral ITAG harvesting	15 (30%)	12 (24%)	14 (28%)
Patients with delayed closure of surgical wound	18 (36%)	16 (32%)	13 (26%)
Patients with multiple incisions	6 (12%)	8 (16%)	13 (26%)

Of the indications that were reimbursed under the NHI, there was generally low alignment with indications reported at respondents’ hospitals or institutions (Table 4). The top indications for which sNPWT was used in hospitals/institutions were: “patients with skin or cutaneous blood flow diseases and disorders that delay wound healing” (36%, n=18) and “patients with diabetes using insulin” (36%, n=18), even though the NHI determines reimbursement eligibility using hemoglobin A1c (HbA1C) count rather than insulin use.

For indications that respondents hoped to see sNPWT expanded to in the future, “patients undergoing emergency surgery” was the most common (32%, n=16), followed by “patients who are undernourished” (which is already an indication reimbursed under the NHI) and “patients with chronic obstructive pulmonary disease (COPD)” (both 30%, n=15), though many other indications were also selected by around a quarter of respondents each (Table 4).

**Figure 5:**
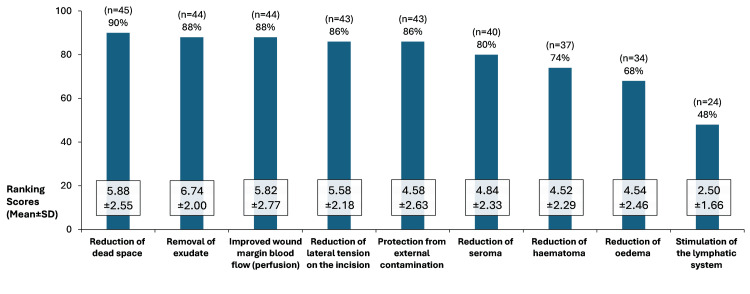
Respondents’ expectations of benefits from sNPWT for SSC prophylaxis and ranking scores for the importance of each benefit among survey respondents (N=50) Footnotes: Respondents were asked: “What benefits do you expect in terms of SSC (including SSI) prevention from using sNPWT prophylactically on closed surgical incisions?” and “please rank the following factors in order of importance for the prophylactic use of sNPWT in the prevention of SSCs, including SSIs, in closed surgical incisions.” Maximum ranking score: 9.00. Abbreviations: SD: standard deviation; sNPWT: single-use negative pressure wound therapy; SSC: surgical site complication; SSC: surgical site infection.

Reduction of dead space was the most expected benefit of sNPWT (90%, n=45) followed by “removal of exudate” and “improved wound margin blood flow” (both 88%, n=44) (Figure 5). The benefits that were most commonly expected from sNPWT also tended to rank highly in terms of importance, with “reduction of dead space” having a ranking score of 5.88 and “removal of exudate” scoring a mean of 6.74 (maximum: 9.00). The least expected benefit was “stimulation of the lymphatic system”, which also had the lowest mean score of 2.50 for importance (Figure 5).

Barriers to sNPWT use

While approximately half of respondents strongly agreed or agreed (44%, n=22) that sNPWT should be used prophylactically after CABG, a substantial portion were neutral or disagreed (Table 5). Similarly, while 52% (n=26) of respondents would strongly recommend or recommend prophylactic sNPWT after CABG for SSC prevention to their colleagues, 30% (n=15) were neutral and 18% (n=9) were against recommending prophylactic sNPWT (Table 5). Of the respondents who were aware of existing consensus and guidelines on prophylactic sNPWT, 69% (n=9/13) strongly agreed or agreed that the available consensus was in support of prophylactic sNPWT after CABG. The Cronbach’s alpha was high (α = 0.91), showing internal consistency and inter-item reliability among the three questions measuring respondents’ strength of agreement on the use of sNPWT.

**Table 5:** Level of agreement with prophylactic sNPWT use after CABG and recommendation rate to colleagues Footnotes: Respondents were asked “Please rate your agreement with the following statement: sNPWT should be used prophylactically in all patients following conventional CABG surgery using open SVG/ITAG harvesting, as a preventative measure for SSCs, including SSIs” and “Please rate your agreement with the following statement: I would recommend the prophylactic use of sNPWT to my colleagues for the prevention of SSCs, including SSIs, following conventional CABG surgery using open SVG/ITAG harvesting.” Abbreviations: CABG: coronary artery bypass grafting; sNPWT: single-use negative pressure wound therapy; SSC: surgical site complication; SVG: saphenous vein graft; SSI: surgical site infection; ITAG: internal thoracic artery graft

	All respondents (N=50)	
	sNPWT should be used prophylactically in all patients after CABG surgery, n(%)	I would recommend sNPWT to my colleagues for SSC prevention after CABG surgery, n(%)
Strongly agree	5 (10%)	6 (12%)
Agree	17 (34%)	20 (40%)
Neural	17 (34%)	15 (30%)
Disagree	8 (16%)	8 (16%)
Strongly disagree	3 (6%)	1 (2%)

**Figure 6:**
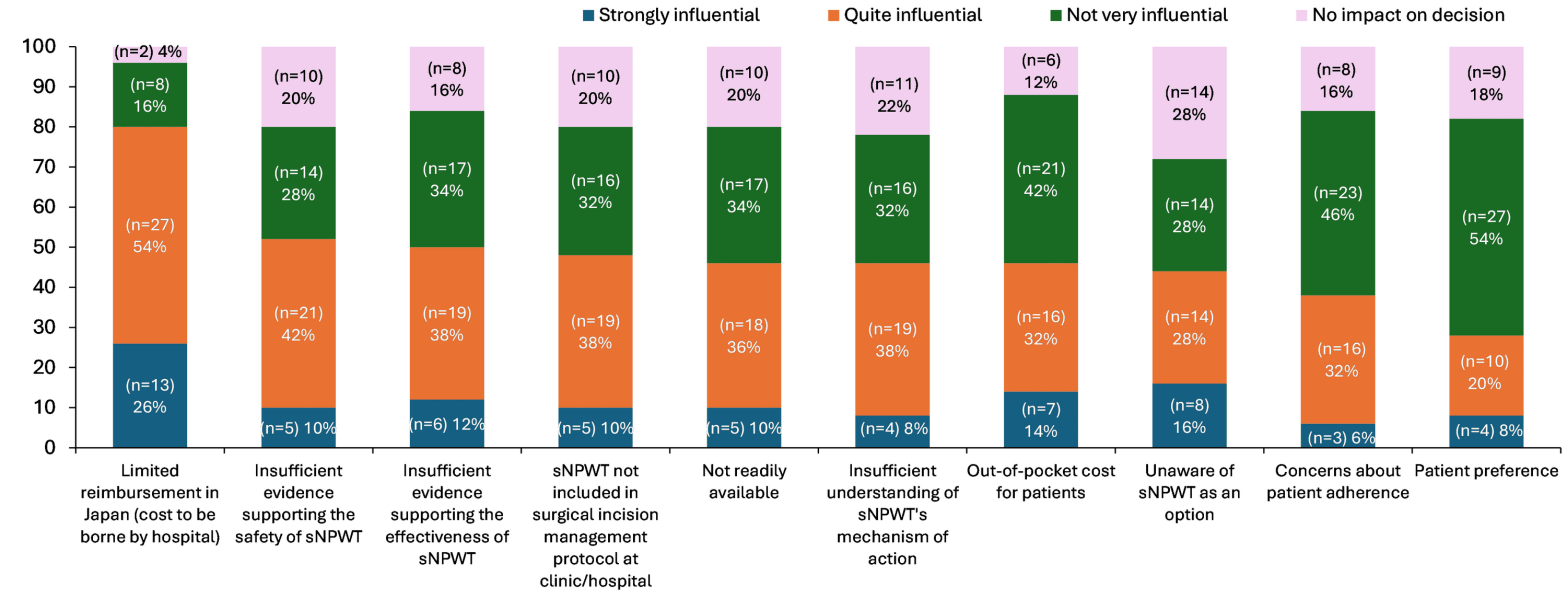
Respondent’s reporting of influential factors for non-use of prophylactic sNPWT after CABG among survey respondents (N=50) Footnotes: Respondents were asked: “Please rate the extent to which each of the following considerations has influenced your past decisions not to use sNPWT prophylactically on your patients’ closed wounds after conventional CABG surgery using open SVG/ITAG harvesting.” Abbreviations: sNPWT: single-use negative pressure wound therapy; CABG: coronary artery bypass grafting; SVG: saphenous vein graft; ITAG: internal thoracic artery graft

**Figure 7:**
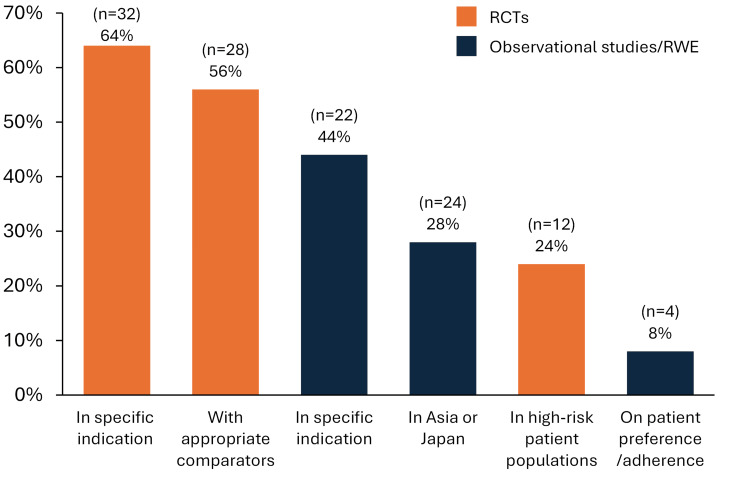
Evidence needs to inform prophylactic sNPWT use after CABG among survey respondents (N=50) Footnote: Respondents were asked to select up to three for “what further clinical evidence would be valuable to inform your prophylactic use of sNPWT after CABG surgery to prevent SSCs, including SSIs?” Abbreviations: CABG: coronary artery bypass grafting; RCT: randomised controlled trial; RWE: real-world evidence, sNPWT: single-use negative pressure wound therapy.

Other than reimbursement (80%, n=40), insufficient evidence for safety (52%, n=26) and effectiveness (50%, n=25) were often cited as reasons for not using prophylactic sNPWT (Figure 6). The majority of respondents indicated that evidence from randomised controlled trials (RCTs), in the specific indication of CV or CABG (64%, n=32) or with appropriate comparators (56%, n=28), was important for informing their clinical decision-making on prophylactic use of sNPWT after CABG (Figure 7). The majority of respondents agreed or strongly agreed with the importance of indication-specific guidelines or consensus (60%, n=30), as well as the need for localised Japan-specific guidelines or consensus (60%, n=30) for encouraging the uptake of prophylactic sNPWT following CABG.

Subgroup analysis by sNPWT use

Demographics were largely similar between respondents with different sNPWT usage patterns/experience (Table 1). There were also no large differences between subgroups in terms of the number of patients with SSCs managed in the past five years (Table 6). However, some trends were observed in sNPWT usage between surgeons from different types of hospitals. While 24% of the overall respondents were from university hospitals, 58% (n=7) of the 12 respondents who used sNPWT routinely were from university hospitals. Conversely, while 38% of the overall respondents were from general hospitals, only 17% (n=2) of those who used sNPWT routinely were from general hospitals. The proportion of surgeons practicing in general hospitals was particularly high (60%, n=6) among never-users of sNPWT.

**Table 6:** Surgeons’ experiences of SSCs management after CABG in the past five years – Subgroup analysis by CV surgeons' prior sNPWT use Footnotes: Respondents were asked “In your practice, how many patients have you managed with SSCs, including SSIs, after CABG surgery for the following incision sites in the past 5 years?” Abbreviations: CABG: coronary bypass grafting; CV: cardiovascular; sNPWT: single-use negative pressure wound therapy; SSC: surgical site infection; SSI: surgical site infection.

	Routine prophylactic users (N=12)	Users only upon signs of SSCs (N=22)	Past sNPWT users (N=6)	sNPWT never users (N=10)
	n (%)	n (%)	n (%)	n (%)
No patients with SSCs				
Median sternotomy	2 (17%)	4 (18%)	2 (33%)	3 (30%)
Lower extremity	1 (8%)	5 (23%)	2 (33%)	3 (30%)
1–5 patients with SSCs				
Median sternotomy	6 (50%)	14 (64%)	1 (17%)	6 (60%)
Lower extremity	6 (50%)	13 (59%)	1 (17%)	5 (50%)
6–10 patients with SSCs				
Median sternotomy	3 (25%)	1 (5%)	1 (17%)	1 (10%)
Lower extremity	3 (25%)	2 (9%)	1 (17%)	2 (20%)
11–20 patients with SSCs				
Median sternotomy	1 (8%)	3 (14%)	1 (17%)	0 (0%)
Lower extremity	2 (17%)	2 (9%)	1 (17%)	0 (0%)
≥21 patients with SSCs				
Median sternotomy	0 (0%)	0 (0%)	1 (17%)	0 (0%)
Lower extremity	0 (0%)	0 (0%)	1 (17%)	0 (0%)

Larger proportions of routine prophylactic users of sNPWT reported that CV surgery had a higher likelihood of SSCs (42%, n=5/12) compared to those who used sNPWT as SSC treatment (23%, n=5/22), past users (33%, n=2/6) and never users (0%, n=0/10). There were no clear differences between the perceived severity of SSC consequences following CV surgeries, compared with other surgery types, between the sNPWT usage subgroups (Table 7).

**Table 7:** Perceptions of SSC risk and severity of consequences by CV surgeons, for different types of CV surgery – Subgroup analysis by CV surgeons’ prior sNPWT use Footnotes: Respondents were asked: “For each of the following cardiovascular surgery types, please indicate if they may be associated with higher likelihood of SSCs or more severe consequences of SSCs, compared with other types of surgery.” Abbreviations: CV: cardiovascular; sNPWT: single-use negative pressure wound therapy; SSC: surgical site complication.

	Routine prophylactic users (N=12)	Users only upon signs of SSCs (N=22)	Past sNPWT users (N=6)	sNPWT never users (N=10)
	n (%)	n (%)	n (%)	n (%)
Perceived SSC risk after CV surgery				
Higher likelihood of SSCs	5 (42%)	5 (23%)	2 (33%)	0 (0%)
Average likelihood of SSCs	3 (25%)	5 (23%)	1 (17%)	6 (60%)
Lower likelihood of SSCs	3 (25%)	12 (55%)	3 (50%)	4 (40%)
Don’t know/no opinion	0 (0%)	0 (0%)	0 (0%)	0 (0%)
Perceived severity of SSC consequences after CV surgery				
More severe consequences	11 (92%)	15 (68%)	4 (67%)	9 (90%)
Consequences are the same	1 (8%)	5 (23%)	2 (33%)	1 (10%)
Less severe consequences	0 (0%)	2 (9%)	0 (0%)	0 (0%)
Don’t know/no opinion	0 (0%)	0 (0%)	0 (0%)	0 (0%)

Existing users of sNPWT were more likely to believe that sNPWT should be used prophylactically after CABG for SSC prevention and recommend prophylactic sNPWT for SSC prevention. Among all of the respondents who would strongly recommend prophylactic sNPWT for SSC prevention after CABG to their colleagues (n=6), four respondents were routine users of prophylactic NPWT, and two respondents were users of sNPWT only upon signs of SSCs. No respondents who were past users or never users of sNPWT indicated they would strongly recommend prophylactic sNPWT following CABG. The three respondents who strongly disagreed with prophylactic sNPWT use after CABG were either past users of sNPWT (n=1) or never users of sNPWT (n=2).

Evidence requirements for clinical decision-making on prophylactic use of sNPWT after CABG differed between subgroups (Table 8). More routine prophylactic users (67%, n=8/12) and users of sNPWT only upon signs of SSCs (73%, n=16/22) reported that high-quality RCTs in the specific indication of CV surgery or CABG were important for clinical decision-making compared with past users (50%, n=3/6) and never users (50%, n=5/10). Instead, greater proportions of never users (90%, n=9/10) responded that appropriate comparators in high-quality RCTs were more important for their clinical decision-making.

**Table 8:** Evidence needs to inform prophylactic sNPWT use after CABG – Subgroup analysis by CV surgeons’ prior sNPWT use Footnote: Respondents were asked to select up to three options for “What further clinical evidence would be valuable to inform your prophylactic use of sNPWT after CABG surgery to prevent SSCs, including SSIs?” Abbreviations: CABG: coronary artery bypass grafting; CV: cardiovascular; RCT: randomised controlled trial; RWE: real-world evidence; sNPWT: single-use negative pressure wound therapy; SSC: surgical site complication; SSI: surgical site infection.

	Routine prophylactic users (N=12)	Users only upon signs of SSCs (N=22)	Past sNPWT users (N=6)	sNPWT never users (N=10)
	n (%)	n (%)	n (%)	n (%)
High-quality RCTs with appropriate comparators	7 (58%)	11 (50%)	1 (17%)	9 (90%)
High-quality RCTs in the specific indication of CV surgery or CABG	8 (67%)	16 (73%)	3 (50%)	5 (50%)
High-quality RCTs in high-risk patient populations in Asia or Japan	1 (8%)	5 (23%)	2 (33%)	4 (40%)
Observational studies providing data on real-world effectiveness of sNPWT on SSC prevention in Asia or Japan	3 (25%)	5 (23%)	2 (33%)	4 (40%)
Observational studies in the specific indication of CV surgery or CABG	6 (50%)	10 (45%)	2 (33%)	4 (40%)
RWE on patient preference and adherence regarding sNPWT	1 (8%)	1 (5%)	1 (17%)	1 (10%)

## Discussion

This market research survey investigated the knowledge, attitudes, and practices of Japanese CV surgeons regarding the use of sNPWT for managing SSCs following CABG. The findings revealed a relatively high awareness of sNPWT but low routine prophylactic use (2%), with key perceived barriers including cost, restrictive reimbursement eligibility criteria, and insufficient evidence. This discussion explores how addressing these perceived barriers could improve postoperative wound management after CABG.

Potential benefits of prophylactic sNPWT use

Several real-world studies have reported the effectiveness of NPWT for reducing SSI incidence [18-20]. For example, a retrospective single-centre study in Japan reported that prophylactic use of NPWT following open-chest cardiac surgery reduced SSI incidence from 10.6% to 2.9% when compared to historical control groups without NPWT [21]. Outside of Japan, an open-label prospective study in Poland showed that NPWT was significantly better at reducing SSIs, and thus increased the likelihood of complication-free wound healing after CABG, compared to SoC [22].

Similarly, a systematic literature review of RCTs comparing NPWT to SoC concluded that there was “moderate-certainty” evidence that NPWT reduces the risk of SSIs, compared to standard dressings, after surgery across indications, including CV surgery [23]. Meta-analyses of RCTs have also demonstrated the effectiveness of sNPWT for significantly reducing the risk of SSCs by over 50%, and for reducing the length of hospital stays by almost half a day when compared to standard dressings [12,13].

Thus, the clinical value of prophylactic sNPWT use following CV surgery must be carefully evaluated further in the context of Japan. Although only 22% of surgeons in this survey reported that they had not encountered an SSC in the past five years, 78% had experienced at least one SSC in their practice. While the reported incidence of deep SWI following CV surgery in Japan, ranging from 1.6% to 1.8% [4,24], is lower than the 2.4% to 4.0% reported in studies from the United States (US) [25,26], SSCs can nevertheless delay recovery and substantially increase healthcare costs.

One study demonstrated up to five times higher postoperative resource use and healthcare costs for patients who experienced an SSI after CV surgery in Japan, compared with those who did not [27]. Furthermore, 80% of surgeons surveyed agreed that SSC prevention following CABG is important. Thus, the prevention of SSCs after CV surgery can be considered of clinical and socioeconomic importance, and the clinical value of prophylactic sNPWT use following CV surgery should be carefully evaluated further in the context of Japan. 

Based on the existing evidence and survey results, there are two key potential benefits of sNPWT. Firstly, prophylactic sNPWT can reduce postoperative complications in high-risk patients by decreasing the risk of SSCs or wound dehiscence and promoting faster recovery. Secondly, sNPWT can assist with more efficient use of healthcare resources by minimising the occurrence of SSIs, leading to cost savings from reductions in prolonged hospital stays, readmissions, reoperations, and additional treatments.

In order to maximise these potential benefits, it is important to improve clinical decision-making by considering strategies to address barriers to usage, such as the complex NHI reimbursement criteria and perceived dearth of evidence, so that the use of prophylactic sNPWT may be prioritised in high-risk patients.

Reimbursement criteria and addressing regional disparities

In this survey, a higher proportion of NPWT users were from university hospitals, who reported higher usage of sNPWT compared with surgeons from general hospitals. In Japan, it is not uncommon for individual facilities to develop their own protocols for SSC prevention and management, which their surgeons must follow. This may have contributed to the variation in NPWT usage patterns across hospital types, which hinders the provision of equitable medical care across facilities of different sizes. As there are no local guidelines or consensus articles on NPWT for SSC prevention after CV surgery in Japan, a national standardised guideline on NPWT use for SSC prevention could help to ensure equitable access and provision of SSC preventative care across hospitals in Japan.

This survey also showed that the reimbursement criteria for sNPWT in Japan may be misaligned with current clinical need. sNPWT is reimbursed by the NHI in Japan only in a small subset of patients classified as high-risk and only in specific care settings [17]. However, these are not aligned with examples of patient characteristics (e.g., COPD, receiving emergency surgery) that have been established as risk factors for SSCs following CV surgery in Japan [24,28]. Survey responses also highlighted the restrictive and complex reimbursement criteria as a key barrier to sNPWT use, suggesting that the current criteria may limit the adoption of sNPWT in general hospitals and regional facilities.

Though Japan’s health technology assessment process factors in decisions already made in other countries such as the United Kingdom (UK) and the US, there are misalignments between UK and Japan reimbursement criteria. In the UK, sNPWT is recommended by the National Institute for Health and Care Excellence (NICE) for closed surgical incisions in patients at high risk of developing SSCs. NICE lists risk factors that healthcare professionals should take into account including age, underlying illness, obesity, smoking, wound classification and site and complexity of the procedure [29]. Contrary to Japan’s NHI, the guidance from NICE does not limit the care settings where patients can or should receive sNPWT [17,29]. This suggests that broader and simpler reimbursement criteria can be implemented cost-effectively for sNPWT.

The benefits of lowered reimbursement barriers include more equitable care delivery, where other clinically relevant high-risk patient groups (e.g. those with COPD or undergoing emergency surgery) can also benefit from sNPWT in Japan. In addition, lowering reimbursement barriers would support the use of sNPWT in regional and general hospitals, which could reduce regional disparities and improve CABG outcomes nationwide.

Japan-specific evidence

According to the survey results, there is a widespread perception among CV surgeons that evidence regarding the safety and efficacy of sNPWT is insufficient. Survey respondents specified that additional RCTs, with appropriate comparators, would be helpful to inform their clinical decision-making. This perception serves as a barrier to the prophylactic use of sNPWT, highlighting a clear need for additional RCTs and observational studies, while increasing awareness of existing evidence.

In addition, cost concerns were cited as a major reason for not using NPWT, which may be relevant at the institutional or individual level. However, evidence suggests that using NPWT can be cost-effective from a payer or societal perspective. NICE guidance reports that sNPWT can be cost-saving in colorectal, cardiothoracic and CV surgery [29]. A German cost-effectiveness study also concluded that sNPWT is cost-saving (by reducing SSCs after CABG) with 100% probability compared to SoC, based on a model which included ICU, intermediate care and general ward care settings [30].

Another cost-effectiveness study from France reported that sNPWT reduced the number of SSCs and was cost-saving by €129,461 among cardiac surgery patients with complications, compared to SoC [18]. However, there are no published cost-effectiveness studies on sNPWT following cardiac surgery in Japan or Asia-Pacific. With the underlying differences in SSI incidence and healthcare funding systems between Japan and other countries, it is essential for further research to evaluate the indications for which sNPWT offers value in Japan.

Finally, the survey findings also highlight the need for education and better awareness among healthcare professionals on the value of sNPWT for SSC management. Among respondents, there was limited familiarity with the preventative benefits of sNPWT, suggesting that targeted training programmes and updates to hospital protocols could help overcome resistance and encourage usage of sNPWT as part of value-based care.

This study was able to survey a group of highly-experienced CV surgeons in Japan and generate insights on their knowledge and perceptions of prophylactic sNPWT following CABG. The survey was also piloted in a smaller sample size and updated to ensure that it was easy to understand and could generate meaningful results. To our knowledge, no other study has been conducted in Japan to assess the knowledge, attitudes and practices of surgeons on prophylactic sNPWT.

However, some limitations to the study should also be noted. While this study is able to provide initial insights, the results are not generalisable as the recruitment approach was not random and no sample size calculations were performed to determine sample size. As the source of recruitment was a proprietary online panel and respondents were enrolled via convenience sampling, there may be some selection bias in the final sample, such as excluding surgeons who are less digitally engaged. The survey was also specific to CABG, which is not the only high-risk CV surgery and thus limits generalisability of the results to the field of CV surgery as a whole. Therefore, results should be interpreted with caution in relation to other high-risk CV surgery types, such as left ventricular assist device implantation. Additionally, the survey relied on the self-report of surgeons’ experience and clinical practices, which led to the possibility of reporting bias. As the survey questions required retrospective recall of current practices and past experiences, answers provided by the respondents may be prone to recall bias [31]. However, as no exposures or outcomes were measured based on the objectives of this survey, recall errors were unlikely to have led to bias, but rather, the lack of recall would have offered insights as to gaps or limitations in respondents’ knowledge or awareness. Non-response bias may also be present in the current study, as respondents who did not consent to participate may have had differing characteristics to those who did consent [32]. Given the recruitment method, we were unable to assess the potential impact of this based on the characteristics of non-respondents. Furthermore, there may have been social desirability bias in the responses to the survey. Respondents may be incentivised to understate the true number of SSCs or SSIs they encounter, as these are adverse outcomes that could be perceived as a reflection of performance or skill [33]. However, as the survey was anonymous and completed online, the risk of social desirability bias was considered minimal. This survey focused on Japanese CV surgeons, with questions tailored to a healthcare practitioner audience. Therefore, insights on patient perspectives in terms of the management of SSCs following CABG, such as the preference for comfort during the wound healing process [34], were not generated. Future studies should consider generating insights on patient preferences regarding sNPWT for SSC management following CABG.

## Conclusions

The survey identified key barriers to the use of prophylactic sNPWT in Japan, including restrictive reimbursement eligibility criteria, cost concerns, insufficient local evidence and low awareness of existing evidence. Addressing these challenges could encourage the prophylactic use of sNPWT in Japan and may lead to reduced postoperative complications and lower healthcare costs, while minimising regional disparities in care.

However, careful consideration of the appropriate indications for sNPWT is needed to ensure clinically valid and cost-effective applications in the real world. Further research focusing on Japan-specific evidence is imperative to establish clear guidelines and optimise the use of sNPWT in post-CABG management.

## Appendices

**Table 9:** Survey on Knowledge, Attitudes and Practices Regarding Prophylactic Single-Use Negative Pressure Wound Therapy After Coronary Artery Bypass Graft Surgery

Questions	Answer Options
Study Introduction	
You are invited to take part in a market research survey of licensed surgeons who perform cardiovascular surgeries in Japan. To allow you to make an informed decision as to whether or not you want to take part in this research, this introduction describes the purpose of the research and the data that will be collected. Taking part in this research is your choice. Please take your time to read the following information carefully.	
What is the purpose of this market research?	
The purpose of this market research is to understand your expert opinion and current practices related to surgical incision management after cardiovascular surgery. The aggregate results from this research will be written into a manuscript for publication in a peer-reviewed journal. As this is a market research survey, it is not intended to collect personal information or reports of adverse events.	
Who is financing and conducting this market research?	
This market research is being managed by Costello Medical Singapore (hereafter referred to as Costello Medical), an independent research consultancy, with the support of All Global, a market research agency. A Study Sponsor is providing financial support to Costello Medical for market research design and analysis, and to All Global for survey translation, distribution and collection of data. Further information on the Study Sponsor will be available at the end of the survey.	
What are my obligations if I take part in this market research?	
If you decide to take part, you will be requested to complete a survey, to the best of your abilities, using a device with internet access. The survey will comprise 35 questions and take approximately 30 minutes to complete.	
What will happen if I take part in this market research?	
If you agree to participate, it is necessary that you read and fully understand this information and provide your informed consent. You will then be able to access the online survey, and your answers will be collected and analysed as part of this market research. The following information will be collected from you: your experiences and current practices regarding surgical incision management and prevention of surgical site complications; your current practices and understanding of negative pressure wound therapy; your expert opinion on the evidence base and clinical consensus regarding negative pressure wound therapy; your demographic characteristics (note that this information will be used for analysis purposes only, and not published). The survey will be open for roughly 3–4 weeks and your answers will be combined with others for analysis and reporting.	
Are there benefits to taking part in this market research?	
There is no direct benefit to you from participating in this market research. The information gained from this research may provide insights on existing practices and opinions in the prevention of surgical site complications following cardiovascular surgery, in order to better understand the role of negative pressure wound therapy in local surgical incision management and help the Study Sponsor ensure that their products meet your clinical needs.	
Are there risks to taking part in this market research?	
There is no anticipated risk associated with taking part in this survey. Participation is entirely voluntary, and your responses will be anonymised.	
How can you opt out of the study?	
Your involvement in this study is entirely voluntary, meaning that you can choose not to take part. You can stop and leave the survey at any time if you decide you no longer want to take part, and the survey will end for you. If you would like to take back your consent after the survey is completed, you can still request for your data to be removed by sending an email to the contact email address provided on your survey invite.	
Will I be paid for taking part in this research?	
Upon completion of the survey, you will be compensated for your time as outlined in your survey invite.	
How will my Information be protected, used and shared?	
During this market research, requested information on your demographics, thoughts, opinions and practices ("Information") will be collected from you. This section describes the protection, use and sharing of your Information. Your privacy is very important, and the Study Sponsor and Investigators use many safeguards to protect your privacy, in accordance with applicable data privacy laws. All Global has been sub-contracted to collect Information submitted by you electronically. Your responses will be anonymised by All Global using an identification (ID) number that is unique to you and not related to or derived from information that identifies you (such as your name or your picture). To maintain anonymity, please do not include personal information or report adverse events in any open-ended responses. Costello Medical and the Study Sponsor’s affiliates, representatives and collaborators will only have access to your anonymised survey data labelled with this ID number for data analysis and interpretation. Your Information may be analysed in any country worldwide. All anonymised Information held by Costello Medical and the Study Sponsor cannot be traced back to you as an individual. Your responses will be combined with other people's Information for analysis. You will not be identified in any materials based on this research to be published in a medical journal or presented at a scientific meeting. Your demographic data (e.g. professional title, age and gender) will not be published. All data from the survey will be stored on All Global’s servers for as long as necessary to fulfil the purposes for which the data are collected. Your survey data may be stored on All Global’s servers for up to 10 years after the survey has been completed. All anonymised data from the survey that are held on Costello Medical’s secure servers will be stored in line with General Data Protection Regulations; this will use opt-in (least privileged) access rights to prevent unauthorised access, and only named individuals involved in this project will be able to access the data. All individual data will be archived from Costello Medical’s server after project completion. If you provide your agreement to participate below, you give permission to the Study Sponsor and Investigators to use and/or share anonymised Information with its affiliates, representatives, collaborators and licensees.	
Screening and Consent	
1. Please indicate “Yes” if you have read the provided information, and consent to taking part in this survey.	□ Yes, I have read and understood the information above about my participation and how my data will be analysed and published. I consent to taking part in this survey.
	□ No, I do not consent to taking part in this survey (screen-out response)
2. Are you a cardiovascular surgeon or cardiac surgeon?	□ Yes
	□ No (screen-out response)
3. Are you licensed and actively practicing in Japan?	□ Yes
	□ No (screen-out response)
4. How many years have you been in practice as a cardiovascular surgeon?	□ 0‒4 years (screen-out response)
	□ 5‒9 years
	□ 10‒15 years
	□ 20 or more years
5. How many coronary artery bypass graft (CABG) surgeries using saphenous vein grafts (SVGs) or internal thoracic artery grafts (ITAGs) have you performed in the past year, as the main operating surgeon? Please provide your best guess if you are unsure of the exact number.	□ 0‒10 per year (screen-out response)
	□ 11‒19 per year (screen-out response)
	□ 20‒29 per year
	□ 30 or more per year
Surgical Site Complications and Incision Management	
In this section we will be asking you some questions regarding your thoughts on, and personal experiences with, surgical site complications (SSCs), as well as your approach to prevention and management of SSCs. SSCs such as dehiscence, seroma, haematoma, fat necrosis or surgical site infections (SSIs) may arise from closed surgical incisions and can lead to delayed healing, poor quality scars or abnormal scarring and decreased quality of life. In rare cases, SSCs can lead to severe consequences, such as deep incisional infections, readmission to the hospital or the intensive care unit (ICU), additional surgical procedures, and increased mortality.	
1. Do you think that patients undergoing cardiovascular surgery, in general, have a higher likelihood of experiencing an SSC, including SSIs, compared with other surgery types?	□ Cardiovascular surgery has a higher likelihood of SSCs
	□ Cardiovascular surgery has an average likelihood of SSCs
	□ Cardiovascular surgery has a lower likelihood of SSCs
	□ Don’t know/no opinion
2. SSCs, including SSIs, may have more severe consequences (e.g. mortality) in some types of surgeries. Do you think SSCs after cardiovascular surgery are more likely to lead to more severe consequences, compared with other surgery types?	□ SSCs after cardiovascular surgery tend to have more severe consequences
	□ Consequences of SSCs after cardiovascular surgery are the same as other surgery types
	□ SSCs after cardiovascular surgery have less severe consequences compared with other types of surgery
	□ Don’t know/no opinion
3. For each of the following cardiovascular surgery types, please indicate if they may be associated with higher likelihood of SSCs or more severe consequences of SSCs, compared with other types of surgery.	
• Conventional CABG	□ Associated with higher likelihood of SSCs
	□ If SSCs occurred, there would be more severe consequences
	□ Neither
	□ Don’t know/no opinion
• CABG with single ITAGs	□ Associated with higher likelihood of SSCs
	□ If SSCs occurred, there would be more severe consequences
	□ Neither
	□ Don’t know/no opinion
• CABG with bilateral ITAGs	□ Associated with higher likelihood of SSCs
	□ If SSCs occurred, there would be more severe consequences
	□ Neither
	□ Don’t know/no opinion
• CABG with skeletonised SVG using full skin incision	□ Associated with higher likelihood of SSCs
	□ If SSCs occurred, there would be more severe consequences
	□ Neither
	□ Don’t know/no opinion
• CABG with skeletonised SVG using bridged skin incision	□ Associated with higher likelihood of SSCs
	□ If SSCs occurred, there would be more severe consequences
	□ Neither
	□ Don’t know/no opinion
• CABG with skeletonised SVG using endoscopic technique	□ Associated with higher likelihood of SSCs
	□ If SSCs occurred, there would be more severe consequences
	□ Neither
	□ Don’t know/no opinion
• CABG with non-touch SVG using full skin incision	□ Associated with higher likelihood of SSCs
	□ If SSCs occurred, there would be more severe consequences
	□ Neither
	□ Don’t know/no opinion
• CABG with non-touch SVG using bridged skin incision	□ Associated with higher likelihood of SSCs
	□ If SSCs occurred, there would be more severe consequences
	□ Neither
	□ Don’t know/no opinion
• CABG with non-touch SVG using endoscopic technique	□ Associated with higher likelihood of SSCs
	□ If SSCs occurred, there would be more severe consequences
	□ Neither
	□ Don’t know/no opinion
• Heart valve surgery	□ Associated with higher likelihood of SSCs
	□ If SSCs occurred, there would be more severe consequences
	□ Neither
	□ Don’t know/no opinion
• Aortic surgery	□ Associated with higher likelihood of SSCs
	□ If SSCs occurred, there would be more severe consequences
	□ Neither
	□ Don’t know/no opinion
• Ventricular assist device implantation	□ Associated with higher likelihood of SSCs
	□ If SSCs occurred, there would be more severe consequences
	□ Neither
	□ Don’t know/no opinion
• Heart transplant	□ Associated with higher likelihood of SSCs
	□ If SSCs occurred, there would be more severe consequences
	□ Neither
	□ Don’t know/no opinion
• Distal bypass surgery	□ Associated with higher likelihood of SSCs
	□ If SSCs occurred, there would be more severe consequences
	□ Neither
	□ Don’t know/no opinion
• Limb amputation	□ Associated with higher likelihood of SSCs
	□ If SSCs occurred, there would be more severe consequences
	□ Neither
	□ Don’t know/no opinion
Note: Where CABG surgery is mentioned, please answer the following questions considering conventional CABG surgery using open SVG/ITAG harvesting.	
4. In your practice, how many patients have you managed with SSCs, including SSIs, after CABG surgery for the following incision sites in the past 5 years?	
• Median sternotomy	□ No patients with SSCs
	□ 1–5 patients with SSCs
	□ 6–10 patients with SSCs
	□ 11–20 patients with SSCs
	□ ≥21 patients with SSCs
• Lower extremity	□ No patients with SSCs
	□ 1–5 patients with SSCs
	□ 6–10 patients with SSCs
	□ 11–20 patients with SSCs
	□ ≥21 patients with SSCs
5. Please rate the importance of the following factors in contributing to the risk of a patient experiencing SSCs after CABG surgery.	
• Pre-operative procedures, such as removal of hair around the surgical site, shower or bath on surgery day, management of nutrition and hydration	□ Not important at all
	□ Somewhat important
	□ Very important
• Intra-operative procedures, such as minimisation of operating room traffic, operating room compliance to hygiene measures, wound irrigation with antiseptic solution or use of skin sealant	□ Not important at all
	□ Somewhat important
	□ Very important
• Postoperative treatment procedures, such as wound dressing choice (e.g. bordered foam dressing, fabric type island dressing, film and pad dressing, hydrocolloid dressing or single-use NPWT)	□ Not important at all
	□ Somewhat important
	□ Very important
• Operative considerations, such as extended surgery time or delayed closure	□ Not important at all
	□ Somewhat important
	□ Very important
• Patients’ clinical characteristics, such as age or presence of comorbidities	□ Not important at all
	□ Somewhat important
	□ Very important
• Patients’ current personal hygiene practices	□ Not important at all
	□ Somewhat important
	□ Very important
• Sternum closure methods and techniques (e.g. continuous suture technique, choice of suture material, amount of tension on closed incision sutures)	□ Not important at all
	□ Somewhat important
	□ Very important
• Standard care bundle for SSIs as per World Health Organization (WHO) or U.S. Centers for Disease Control and Prevention (CDC) recommendations	□ Not important at all
	□ Somewhat important
	□ Very important
6. Please indicate your level of agreement with the following statement: During the post-surgery period, prevention of SSCs, including SSIs is a priority in managing patients who have undergone CABG surgery.	□ Strongly disagree
	□ Disagree
	□ Neutral
	□ Agree
	□ Strongly agree
7. Which of the following strategies do you currently use to prevent the occurrence of SSCs, including SSIs in your patients, after CABG surgery?	
• Antibiotic prophylaxis	□ Never
	□ Occasionally
	□ Sometimes
	□ Often
	□ Always
• Negative pressure wound therapy	□ Never
	□ Occasionally
	□ Sometimes
	□ Often
	□ Always
• Single-use negative pressure wound therapy	□ Never
	□ Occasionally
	□ Sometimes
	□ Often
	□ Always
• Bordered foam dressing	□ Never
	□ Occasionally
	□ Sometimes
	□ Often
	□ Always
• Film and pad dressing	□ Never
	□ Occasionally
	□ Sometimes
	□ Often
	□ Always
• Hydrocolloid dressing	□ Never
	□ Occasionally
	□ Sometimes
	□ Often
	□ Always
• Composite dressing	□ Never
	□ Occasionally
	□ Sometimes
	□ Often
	□ Always
• Subcutaneous drains	□ Never
	□ Occasionally
	□ Sometimes
	□ Often
	□ Always
• Antimicrobial sutures	□ Never
	□ Occasionally
	□ Sometimes
	□ Often
	□ Always
• Adhesive dressing (glue)	□ Never
	□ Occasionally
	□ Sometimes
	□ Often
	□ Always
• Other closure dressing (please specify)	□ Never
	□ Occasionally
	□ Sometimes
	□ Often
	□ Always
8. Please rate the importance of the following considerations when deciding on a strategy for preventing SSCs, including SSIs, following CABG surgery.	
• Risk profile: patients’ age	□ Not important at all
	□ Somewhat important
	□ Very important
• Risk profile: patients’ comorbidities	□ Not important at all
	□ Somewhat important
	□ Very important
• Risk profile: surgery characteristics (e.g. incision site, delayed closure)	□ Not important at all
	□ Somewhat important
	□ Very important
• Overall monetary cost	□ Not important at all
	□ Somewhat important
	□ Very important
• Out-of-pocket cost to patient	□ Not important at all
	□ Somewhat important
	□ Very important
• Cost to the hospital	□ Not important at all
	□ Somewhat important
	□ Very important
• Patients’ preference (e.g. based on their lifestyle and daily routines)	□ Not important at all
	□ Somewhat important
	□ Very important
• Other (please specify)	□ Not important at all
	□ Somewhat important
	□ Very important
Single-Use Negative Pressure Wound Therapy	
In this section we will ask you questions related to your understanding of single-use negative pressure wound therapy (sNPWT) in Japan. We will then also ask you questions regarding your opinions on the use of sNPWT, and on your current use of sNPWT, if any.	
9. Are you familiar with sNPWT and what it is used for?	□ Very familiar
	□ Somewhat familiar
	□ Not at all familiar
	□ Don’t know/not sure
NPWT provides controlled suction to create negative pressure over a wound. This is powered by a vacuum pump, which is typically covered by foam or gauze and adhesive film. Traditionally, NPWT systems also have a canister to collect fluids removed from the wound. NPWT systems can be used to reduce the incidence of superficial and deep incisional SSCs, including SSIs on closed surgical incisions. sNPWT are portable single-use systems, often with a disposable pump and ready-made dressings, which are typically used for a week. They can be battery-operated and are a more accessible option for NPWT due to their size and ease of use.	
10. Based on your understanding, please select all indications for which sNPWT is approved for use in Japan. (Respondents must select all correct options and no incorrect options to score 1 point.)	□ Hard-to-heal open wounds (correct)
	□ Closed surgical wounds in patients at high SSI risk for prophylactic purposes (correct)
	□ Closed surgical wounds to treat SSCs
	□ Traumatic wounds
	□ None of the above
11. sNPWT was approved in Japan for prophylactic use in closed surgical wounds in patients at high SSI risk in 2019. However, Japan reimbursement criteria specify that sNPWT will be reimbursed for closed surgical wounds under the National Health Insurance (NHI) system only in a subset of “high-risk” patients, and only in specific care settings. Based on your understanding, please select the settings in which sNPWT may be reimbursed. (Respondents must select all correct options and no incorrect options to score 1 point.)	□ General ward
	□ Emergency treatment
	□ ICU (correct)
	□ High care unit (correct)
	□ Stroke care unit (correct)
	□ Paediatric ICU (correct)
	□ Neonatal ICU (correct)
	□ Perinatal ICU (correct)
12. From the following indications, please select all that apply for prophylactic use of sNPWT. (For “indication for sNPWT covered by NHI reimbursement”, respondents must select more correct options than incorrect to score 1 point.)	
• Patients above the age of 65	□ Indication for sNPWT covered by NHI reimbursement
	□ Indication for sNPWT at my own hospital or institution
	□ Indication that I hope to see sNPWT expanded to in the future
	□ Not a suitable indication for sNPWT
	□ Not applicable/don’t know
• Patients above the age of 80	□ Indication for sNPWT covered by NHI reimbursement
	□ Indication for sNPWT at my own hospital or institution
	□ Indication that I hope to see sNPWT expanded to in the future
	□ Not a suitable indication for sNPWT
	□ Not applicable/don’t know
• Patients with obesity (BMI ≥30) (correct)	□ Indication for sNPWT covered by NHI reimbursement
	□ Indication for sNPWT at my own hospital or institution
	□ Indication that I hope to see sNPWT expanded to in the future
	□ Not a suitable indication for sNPWT
	□ Not applicable/don’t know
• Patients who are underweight (BMI <18)	□ Indication for sNPWT covered by NHI reimbursement
	□ Indication for sNPWT at my own hospital or institution
	□ Indication that I hope to see sNPWT expanded to in the future
	□ Not a suitable indication for sNPWT
	□ Not applicable/don’t know
• Patients who are undernourished (correct)	□ Indication for sNPWT covered by NHI reimbursement
	□ Indication for sNPWT at my own hospital or institution
	□ Indication that I hope to see sNPWT expanded to in the future
	□ Not a suitable indication for sNPWT
	□ Not applicable/don’t know
• Patients with diabetes (HbA1C count ≥6.6%) (correct)	□ Indication for sNPWT covered by NHI reimbursement
	□ Indication for sNPWT at my own hospital or institution
	□ Indication that I hope to see sNPWT expanded to in the future
	□ Not a suitable indication for sNPWT
	□ Not applicable/don’t know
• Patients with diabetes using insulin	□ Indication for sNPWT covered by NHI reimbursement
	□ Indication for sNPWT at my own hospital or institution
	□ Indication that I hope to see sNPWT expanded to in the future
	□ Not a suitable indication for sNPWT
	□ Not applicable/don’t know
• Patients with chronic kidney disease	□ Indication for sNPWT covered by NHI reimbursement
	□ Indication for sNPWT at my own hospital or institution
	□ Indication that I hope to see sNPWT expanded to in the future
	□ Not a suitable indication for sNPWT
	□ Not applicable/don’t know
• Patients on dialysis (correct)	□ Indication for sNPWT covered by NHI reimbursement
	□ Indication for sNPWT at my own hospital or institution
	□ Indication that I hope to see sNPWT expanded to in the future
	□ Not a suitable indication for sNPWT
	□ Not applicable/don’t know
• Patients who are current smokers	□ Indication for sNPWT covered by NHI reimbursement
	□ Indication for sNPWT at my own hospital or institution
	□ Indication that I hope to see sNPWT expanded to in the future
	□ Not a suitable indication for sNPWT
	□ Not applicable/don’t know
• Patients with chronic obstructive pulmonary disease	□ Indication for sNPWT covered by NHI reimbursement
	□ Indication for sNPWT at my own hospital or institution
	□ Indication that I hope to see sNPWT expanded to in the future
	□ Not a suitable indication for sNPWT
	□ Not applicable/don’t know
• Patients on steroid based treatments (correct)	□ Indication for sNPWT covered by NHI reimbursement
	□ Indication for sNPWT at my own hospital or institution
	□ Indication that I hope to see sNPWT expanded to in the future
	□ Not a suitable indication for sNPWT
	□ Not applicable/don’t know
• Patients who are immunocompromised (correct)	□ Indication for sNPWT covered by NHI reimbursement
	□ Indication for sNPWT at my own hospital or institution
	□ Indication that I hope to see sNPWT expanded to in the future
	□ Not a suitable indication for sNPWT
	□ Not applicable/don’t know
• Patients with dementia	□ Indication for sNPWT covered by NHI reimbursement
	□ Indication for sNPWT at my own hospital or institution
	□ Indication that I hope to see sNPWT expanded to in the future
	□ Not a suitable indication for sNPWT
	□ Not applicable/don’t know
• Patients on blood-thinning medications	□ Indication for sNPWT covered by NHI reimbursement
	□ Indication for sNPWT at my own hospital or institution
	□ Indication that I hope to see sNPWT expanded to in the future
	□ Not a suitable indication for sNPWT
	□ Not applicable/don’t know
• Patients with skin or cutaneous blood flow diseases and disorders that delay wound healing (correct)	□ Indication for sNPWT covered by NHI reimbursement
	□ Indication for sNPWT at my own hospital or institution
	□ Indication that I hope to see sNPWT expanded to in the future
	□ Not a suitable indication for sNPWT
	□ Not applicable/don’t know
• Patients with a history of SSCs	□ Indication for sNPWT covered by NHI reimbursement
	□ Indication for sNPWT at my own hospital or institution
	□ Indication that I hope to see sNPWT expanded to in the future
	□ Not a suitable indication for sNPWT
	□ Not applicable/don’t know
• Patients undergoing re-surgery at the same site as a prior surgery (correct)	□ Indication for sNPWT covered by NHI reimbursement
	□ Indication for sNPWT at my own hospital or institution
	□ Indication that I hope to see sNPWT expanded to in the future
	□ Not a suitable indication for sNPWT
	□ Not applicable/don’t know
• Patients undergoing emergency surgery	□ Indication for sNPWT covered by NHI reimbursement
	□ Indication for sNPWT at my own hospital or institution
	□ Indication that I hope to see sNPWT expanded to in the future
	□ Not a suitable indication for sNPWT
	□ Not applicable/don’t know
• Patients with bilateral ITAG harvesting	□ Indication for sNPWT covered by NHI reimbursement
	□ Indication for sNPWT at my own hospital or institution
	□ Indication that I hope to see sNPWT expanded to in the future
	□ Not a suitable indication for sNPWT
	□ Not applicable/don’t know
• Patients with delayed closure of surgical wound	□ Indication for sNPWT covered by NHI reimbursement
	□ Indication for sNPWT at my own hospital or institution
	□ Indication that I hope to see sNPWT expanded to in the future
	□ Not a suitable indication for sNPWT
	□ Not applicable/don’t know
• Patients with multiple incisions	□ Indication for sNPWT covered by NHI reimbursement
	□ Indication for sNPWT at my own hospital or institution
	□ Indication that I hope to see sNPWT expanded to in the future
	□ Not a suitable indication for sNPWT
	□ Not applicable/don’t know
Current Practice and Perceptions of sNPWT	
In the following section, we will be asking questions related to your perceptions of sNPWT, and on your current use of sNPWT, if any. As a reminder, please do not report any personal information or specific adverse events in your responses to this survey.	
13. Have you ever used sNPWT on closed surgical wounds after conventional CABG with open SVG/ITAG harvesting?	□ Yes, I currently use sNPWT routinely as a prophylactic measure
	□ Yes, I currently use sNPWT only when there is a sign of an SSC
	□ Yes, I have used sNPWT in the past but do not currently use it
	□ No, I have never used sNPWT
14. (Show only if “Yes, I have used sNPWT in the past but do not currently use it” selected in Question 13) Why did you stop using sNPWT?	□ High cost
	□ Limited reimbursement
	□ Adverse events
	□ Patient preference
	□ Patient adherence issues
	□ Lack of absorbency
	□ Lack of ability to stay secured in place
	□ Lack of effectiveness
	□ No (or limited) patients with SSCs
	□ Other (please specify)
15. (Show only if “No, I have never used sNPWT” selected in Question 13) Why have you never used sNPWT?	□ Not aware of sNPWT
	□ sNPWT is not readily available to me in my practice
	□ Not convinced of effectiveness (e.g. lack of evidence)
	□ Not convinced of safety (e.g. lack of evidence)
	□ High cost
	□ Limited reimbursement
	□ Patient preference
	□ Concerns regarding patient adherence
	□ Other (please specify)
16. Have you ever used sNPWT prophylactically for SSC prevention in other surgery types?	□ No
	□ Yes (please specify surgery type)
17. What benefits do you expect in terms of SSC (including SSI) prevention from using sNPWT prophylactically on closed surgical incisions? Please select “Yes” if it is an effect or benefit you expect from sNPWT and “No” if you do not expect it.	
• Protection from external contamination	□ Yes
	□ No
• Stimulation of the lymphatic system	□ Yes
	□ No
• Reduction of oedema	□ Yes
	□ No
• Reduction of seroma	□ Yes
	□ No
• Reduction of haematoma	□ Yes
	□ No
• Improved wound margin blood flow (perfusion)	□ Yes
	□ No
• Removal of exudate	□ Yes
	□ No
• Reduction of lateral tension on the incision	□ Yes
	□ No
• Reduction of dead space	□ Yes
	□ No
18. Please rank the following factors in order of importance for the prophylactic use of sNPWT in the prevention of SSCs, including SSIs, in closed surgical incisions.	Rank from most to least important:
	• Protection from external contamination
	• Stimulation of the lymphatic system
	• Reduction of oedema
	• Reduction of seroma
	• Reduction of haematoma
	• Improving wound margin blood flow (perfusion)
	• Removal of exudate
	• Reduction of lateral tension on the incision
	• Reduction of dead space
19. Please rate your agreement with the following statement: sNPWT should be used prophylactically in all patients following conventional CABG surgery using open SVG/ITAG harvesting, as a preventative measure for SSCs, including SSIs.	□ Strongly agree
	□ Agree
	□ Neither agree nor disagree
	□ Disagree
	□ Strongly disagree
20. Please rate the extent to which each of the following considerations has influenced your past decisions not to use sNPWT prophylactically on your patients’ closed wounds after conventional CABG surgery using open SVG/ITAG harvesting:	
• Unaware of sNPWT as an option	□ Strongly influential
	□ Quite influential
	□ Not very influential
	□ No impact on decision
• Insufficient understanding of sNPWT’s mechanism of action	□ Strongly influential
	□ Quite influential
	□ Not very influential
	□ No impact on decision
• Insufficient evidence supporting the effectiveness of sNPWT	□ Strongly influential
	□ Quite influential
	□ Not very influential
	□ No impact on decision
• Insufficient evidence supporting the safety of sNPWT	□ Strongly influential
	□ Quite influential
	□ Not very influential
	□ No impact on decision
• sNPWT not included in surgical incision management protocol at clinic/hospital	□ Strongly influential
	□ Quite influential
	□ Not very influential
	□ No impact on decision
• Not readily available	□ Strongly influential
	□ Quite influential
	□ Not very influential
	□ No impact on decision
• Limited reimbursement in Japan (cost to be borne by hospital)	□ Strongly influential
	□ Quite influential
	□ Not very influential
	□ No impact on decision
• Out-of-pocket cost for patients	□ Strongly influential
	□ Quite influential
	□ Not very influential
	□ No impact on decision
• Concerns about patient adherence	□ Strongly influential
	□ Quite influential
	□ Not very influential
	□ No impact on decision
• Patient preference	□ Strongly influential
	□ Quite influential
	□ Not very influential
	□ No impact on decision
21. Please rate your agreement with the following statement: I would recommend the prophylactic use of sNPWT to my colleagues for the prevention of SSCs, including SSIs, following conventional CABG surgery using open SVG/ITAG harvesting.	□ Strongly agree
	□ Agree
	□ Neither agree nor disagree
	□ Disagree
	□ Strongly disagree
Evidence Base and Current Consensus on sNPWT	
In the following section, we will ask you about the evidence available for sNPWT, as well as the current clinical guidelines/expert consensus on NPWT (traditional or single-use), and the extent to which these affect your clinical decision-making. Where CABG surgery is mentioned, please answer the following questions considering conventional CABG surgery using open SVG/ITAG harvesting.	
22. Do you believe that there is currently sufficient high-quality clinical evidence to demonstrate the effectiveness of prophylactic sNPWT on closed surgical wounds following CABG surgery for the prevention of SSCs, including SSIs, in your patients?	□ Yes
	□ No
	□ Unsure
23. What further clinical evidence would be valuable to inform your prophylactic use of sNPWT after CABG surgery to prevent SSCs, including SSIs? (please select up to 3 categories of highest priority)	□ High-quality RCTs with appropriate comparators (e.g. antibiotic prophylaxis, or other standard of care in Japan)
	□ High-quality RCTs of sNPWT in the specific indication of cardiovascular surgery or CABG surgery
	□ High-quality RCTs of sNPWT in high-risk patient populations, such as dialysis patients or elderly patients in Asia or Japan
	□ Observational studies providing data on real-world effectiveness of sNPWT on SSC prevention in Asia or Japan
	□ Observational studies on sNPWT used in the specific indication of cardiovascular surgery or CABG surgery
	□ Real-world evidence on patient preference and adherence regarding sNPWT
	□ Other (please specify)
	□ Don’t know/unsure
24. Please rate your agreement with the following statements:	
• Clinical data from other surgery types in Japan (e.g. obstetrics, gastrointestinal or orthopaedics) is appropriate to inform decisions on the prophylactic use of sNPWT in my patients, after CABG surgery	□ Strongly agree
	□ Agree
	□ Neither agree nor disagree
	□ Disagree
	□ Strongly disagree
• Clinical data on cardiovascular surgery from other geographies is appropriate to inform decisions on the prophylactic use of sNPWT in my patients after CABG surgery	□ Strongly agree
	□ Agree
	□ Neither agree nor disagree
	□ Disagree
	□ Strongly disagree
• Clinical data from other surgery types and other geographies is appropriate to inform decisions on the prophylactic use of sNPWT in my patients after CABG surgery in Japan	□ Strongly agree
	□ Agree
	□ Neither agree nor disagree
	□ Disagree
	□ Strongly disagree
25. Are you familiar with any guidelines or expert consensus (local or international) on prophylactic (s)NPWT use for closed surgical wounds following CABG surgery?	□ Yes
	□ No
	□ Don’t know/not sure
26. (Show only if “Yes” chosen in question 25) Please rate your agreement with the following statement: Current guidelines and/or expert consensus support the prophylactic use of sNPWT on closed surgical wounds following CABG surgery.	□ Strongly agree
	□ Agree
	□ Neither agree nor disagree
	□ Disagree
	□ Strongly disagree
A few international guidelines and consensus statements have addressed the potential role of NPWT in closed surgical incision management. In particular, the World Union of Wound Healing Society (WUWHS) has published a 2016 consensus article proposing that NPWT should be used prophylactically in closed surgical incisions for patients who have “intrinsic risk factors” for SSCs and patients who are undergoing “a surgical procedure associated with higher incidence and/or higher consequence of surgical site complications”.	
27. Would the above recommendation impact your clinical decision to use sNPWT prophylactically in your patients’ closed surgical wounds, following CABG surgery?	□ I am more likely to use it
	□ I am less likely to use it
	□ No impact
	□ Don’t know/Unsure
28. (Show only if “No impact” chosen in question 27) Why does the WUWHS recommendation have no impact on your clinical decision to use sNPWT prophylactically following CABG surgery?	□ I personally disagree with the recommendation
	□ International consensus may not be applicable to the Japan context
	□ Recommendation is from too many years ago and may be based on outdated evidence
	□ The recommendation is not applicable to CABG surgery
	□ Other (please specify)
29. Would the above recommendation impact your clinical decision to use sNPWT prophylactically in your patients’ closed surgical wounds following CABG surgery?	□ I am more likely to use it
	□ I am less likely to use it
	□ No impact
	□ Don’t know/Unsure
30. (Show only if “No impact” chosen in question 29) Why does the JSSI recommendation have no impact on your clinical decision to use sNPWT prophylactically following CABG surgery?	□ I personally disagree with the statement
	□ The statement does not make any clear recommendation
	□ Recommendation is from too many years ago and may be based on outdated evidence
	□ The recommendation is not applicable to CABG surgery
	□ I do not consider SSI risk in my clinical decision-making
	□ Other (please specify)
31. Please rate your agreement with the following statements:	
• I would be more likely to use sNPWT prophylactically for closed surgical wounds following CABG surgery, if a Japanese expert body made a recommendation specific to this indication as part of a guideline or consensus article.	□ Strongly agree
	□ Agree
	□ Neither agree nor disagree
	□ Disagree
	□ Strongly disagree
• Local Japanese guidelines on prophylactic use of sNPWT are needed as they would take into consideration the Japan-specific context (e.g. local SSC and SSI rates, reimbursement/approval status) that are not considered in international guidelines.	□ Strongly agree
	□ Agree
	□ Neither agree nor disagree
	□ Disagree
	□ Strongly disagree
• Indication-specific guidelines are needed on the prophylactic use of sNPWT, as cardiovascular surgery has specific considerations (e.g. higher consequences of SSCs) compared with other surgery types.	□ Strongly agree
	□ Agree
	□ Neither agree nor disagree
	□ Disagree
	□ Strongly disagree
Demographic Characteristics	
We have a few final questions for this last section of the survey.	
32. How old are you (as of your last birthday)?	□ <29 years
	□ 30–39 years
	□ 40–49 years
	□ 50–59 years
	□ ≥60 years
33. Please indicate your gender.	□ Male
	□ Female
	□ Other
	□ Prefer not to say
34. What is your professional role?	□ Chair of department
	□ Director of department
	□ Chief Physician
	□ Senior Physician
	□ Associate Physician
	□ Senior Consultant
	□ Senior Resident
	□ Consultant
	□ Physician
	□ Resident
	□ Other
35. What type of hospital are you affiliated with?	□ University
	□ National/Public
	□ Other Public
	□ General
	□ Other (please specify)
End of Survey	
You have reached the end of the survey. Thank you for taking the time to provide your valuable thoughts and insights for this study. This market research is being financed by Smith & Nephew PLC (hereafter referred to as Smith & Nephew). Smith & Nephew is providing financial support to Costello Medical for market research design and analysis, and to All Global for survey translation, distribution and collection of data. Smith & Nephew’s privacy policy is available at this link: https://www.smith-nephew.com/ja-jp/privacy-policy.	

## References

[1] (2025). Closed surgical incision management: understanding the role of NPWT. https://woundsinternational.com/consensus-documents/consensus-document-closed-surgical-incision-management-understanding-the-role-of-npwt-wme/.

[2] Biancari F, Santoro G, Provenzano F (2022). Negative-pressure wound therapy for prevention of sternal wound infection after adult cardiac surgery: systematic review and meta-analysis. J Clin Med.

[3] Song Y, Chu W, Sun J, Liu X, Zhu H, Yu H, Shen C (2023). Review on risk factors, classification, and treatment of sternal wound infection. J Cardiothorac Surg.

[4] Hirahara N, Miyata H, Motomura N, Kohsaka S, Nishimura T, Takamoto S (2020). Procedure- and hospital-level variation of deep sternal wound infection from All-Japan Registry. Ann Thorac Surg.

[5] Oosterlinck W, Algoet M, Balkhy HH (2023). Minimally invasive coronary surgery: how should it be defined?. Innovations (Phila).

[6] Gulack BC, Kirkwood KA, Shi W (2018). Secondary surgical-site infection after coronary artery bypass grafting: a multi-institutional prospective cohort study. J Thorac Cardiovasc Surg.

[7] Hassoun-Kheir N, Hasid I, Bozhko M, Shaban Z, Glam R, Hussein K, Paul M (2018). Risk factors for limb surgical site infection following coronary artery bypass graft using open great saphenous vein harvesting: a retrospective cohort study. Interact Cardiovasc Thorac Surg.

[8] Saito A, Motomura N, Kumamaru H, Miyata H, Arai H (2023). Annual report for 2019 by the Japanese Association for Coronary Artery Surgery. Ann Thorac Cardiovasc Surg.

[9] Shih T, Zhang M, Kommareddi M (2014). Center-level variation in infection rates after coronary artery bypass grafting. Circ Cardiovasc Qual Outcomes.

[10] Fiocco A, Dini M, Lorenzoni G, Gregori D, Colli A, Besola L (2024). The prophylactic use of negative-pressure wound therapy after cardiac surgery: a meta-analysis. J Hosp Infect.

[11] (2025). Surgical wound dehiscence: improving prevention and outcomes. https://woundsinternational.com/consensus-documents/surgical-wound-dehiscence-improving-prevention-and-outcomes/.

[12] Groenen H, Jalalzadeh H, Buis DR (2023). Incisional negative pressure wound therapy for the prevention of surgical site infection: an up-to-date meta-analysis and trial sequential analysis. EClinicalMedicine.

[13] Leaper D, Strugala Strugala, V V (2018). The benefit of PICO single use NPWT system to reduce surgical site complications: summary of a meta-analysis with implications for clinical practice. Wounds Int.

[14] Scalise A, Calamita R, Tartaglione C (2016). Improving wound healing and preventing surgical site complications of closed surgical incisions: a possible role of incisional negative pressure wound therapy. A systematic review of the literature. Int Wound J.

[15] (2025). Global guidelines for the prevention of surgical site infection. https://www.who.int/publications/i/item/9789241550475.

[16] (2025). The pharmaceuticals and medical devices agency annual report FY 2014. https://www.pmda.go.jp/files/000208305.pdf.

[17] (2025). New medical devices approved in FY 2019. https://www.pmda.go.jp/files/000244014.pdf.

[18] Tabley A, Aludaat C, Le Guillou V (2020). A survey of cardiac surgery infections with PICO negative pressure therapy in high-risk patients. Ann Thorac Surg.

[19] Chan KS, Arunaachalam M, Hong Q (2020). Outcomes of incisional negative pressure wound therapy following brachiobasilic transposition arteriovenous fistula creation: a 1:2 propensity score matched study. Int Wound J.

[20] Rodden D, Taylor A (2015). NPWT: Incision Management in High Risk Cardiothoracic Patients - Reducing Surgical Site Infection and Length of Stay. Poster presented at Wounds UK.

[21] Kurazumi H, Suzuki R, Nawata R (2022). Feasibility of open chest management with modified negative pressure wound therapy immediately after cardiac surgery. Interact Cardiovasc Thorac Surg.

[22] Witt-Majchrzak A, Żelazny P, Snarska J (2015). Preliminary outcome of treatment of postoperative primarily closed sternotomy wounds treated using negative pressure wound therapy. Pol Przegl Chir.

[23] Norman G, Shi C, Goh EL (2022). Negative pressure wound therapy for surgical wounds healing by primary closure. Cochrane Database Syst Rev.

[24] Kubota H, Miyata H, Motomura N (2013). Deep sternal wound infection after cardiac surgery. J Cardiothorac Surg.

[25] Bonacchi M, Prifti E, Bugetti M (2018). Deep sternal infections after in situ bilateral internal thoracic artery grafting for left ventricular myocardial revascularization: predictors and influence on 20-year outcomes. J Thorac Dis.

[26] Gaudino M, Audisio K, Rahouma M (2023). Association between sternal wound complications and 10-year mortality following coronary artery bypass grafting. J Thorac Cardiovasc Surg.

[27] Kobayashi J, Kusachi S, Sawa Y (2015). Socioeconomic effects of surgical site infection after cardiac surgery in Japan. Surg Today.

[28] Tatsuishi W, Yamamoto H, Nakai M, Tanemoto K, Miyata H, Motomura N (2022). Incidence and outcomes of surgical site infection after cardiovascular surgery (complete republication). Gen Thorac Cardiovasc Surg.

[29] (2025). PICO negative pressure wound dressings for closed surgical incisions, medical technologies guidance [MTG43]. https://www.nice.org.uk/guidance/mtg43.

[30] Nherera LM, Trueman P, Schmoeckel M, Fatoye FA (2018). Cost-effectiveness analysis of single use negative pressure wound therapy dressings (sNPWT) compared to standard of care in reducing surgical site complications (SSC) in patients undergoing coronary artery bypass grafting surgery. J Cardiothorac Surg.

[31] Althubaiti A (2016). Information bias in health research: definition, pitfalls, and adjustment methods. J Multidiscip Healthc.

[32] Davern M (2013). Nonresponse rates are a problematic indicator of nonresponse bias in survey research. Health Serv Res.

[33] Tanner J, Brierley Jones L, Rochon M (2023). Barriers and facilitators for surgical site infection surveillance for adult cardiac surgery in a high-income setting: an in-depth exploration. J Hosp Infect.

[34] Timmermans FW, Mokken SE, Smit JM, Bouman MB, van de Grift TC, Mullender MG, Middelkoop E (2022). The impact of incisional negative pressure wound therapy on scar quality and patient-reported outcomes: a within-patient-controlled, randomised trial. Wound Repair Regen.

